# Genetic heterogeneity of within-family variance of body weight in Atlantic salmon (*Salmo salar*)

**DOI:** 10.1186/1297-9686-45-41

**Published:** 2013-10-17

**Authors:** Anna K Sonesson, Jørgen Ødegård, Lars Rönnegård

**Affiliations:** 1 Nofima AS, Post Box 210, Ås 1431, Norway; 2 Dalarna University, 791 88 Falun, Sweden

## Abstract

**Background:**

Canalization is defined as the stability of a genotype against minor variations in both environment and genetics. Genetic variation in degree of canalization causes heterogeneity of within-family variance. The aims of this study are twofold: (1) quantify genetic heterogeneity of (within-family) residual variance in Atlantic salmon and (2) test whether the observed heterogeneity of (within-family) residual variance can be explained by simple scaling effects.

**Results:**

Analysis of body weight in Atlantic salmon using a double hierarchical generalized linear model (DHGLM) revealed substantial heterogeneity of within-family variance. The 95% prediction interval for within-family variance ranged from ~0.4 to 1.2 kg^2^, implying that the within-family variance of the most extreme high families is expected to be approximately three times larger than the extreme low families. For cross-sectional data, DHGLM with an animal mean sub-model resulted in severe bias, while a corresponding sire-dam model was appropriate. Heterogeneity of variance was not sensitive to Box-Cox transformations of phenotypes, which implies that heterogeneity of variance exists beyond what would be expected from simple scaling effects.

**Conclusions:**

Substantial heterogeneity of within-family variance was found for body weight in Atlantic salmon. A tendency towards higher variance with higher means (scaling effects) was observed, but heterogeneity of within-family variance existed beyond what could be explained by simple scaling effects. For cross-sectional data, using the animal mean sub-model in the DHGLM resulted in biased estimates of variance components, which differed substantially both from a standard linear mean animal model and a sire-dam DHGLM model. Although genetic differences in canalization were observed, selection for increased canalization is difficult, because there is limited individual information for the variance sub-model, especially when based on cross-sectional data. Furthermore, potential macro-environmental changes (diet, climatic region, etc.) may make genetic heterogeneity of variance a less stable trait over time and space.

## Background

In livestock and aquaculture production systems, individuals that can perform well in different environments and against different background genetics are preferred. In evolutionary genetics, the term canalization describes the stability or buffering ability of a genotype against minor variations, not only in the environment but also in the genetic make-up [1].

In animal breeding, genetic differences in phenotypic stability can be detected as genetic heterogeneity of (within-family) residual variance, where a low variance indicates stable performance across environmental factors. Genetic heterogeneity of residual variance has been estimated for different traits in a number of land-based species, e.g., litter size in sheep [2], pigs [3], mice [4] and rabbits [5] and weight in piglets [6] and broilers [7] (review of Hill and Mulder [8]) and more recently somatic cell scores and milk yield in dairy cattle [9] and body weight in rainbow trout [10]. Overall, these studies show substantial genetic heterogeneity of (within-family) residual variance but heritability of individual dispersion as defined in [11] is usually low (<0.05). This is due to the fact that the dispersion parameters of an individual cannot be measured directly but are inferred through estimated residual(s) (i.e., deviations from the expected mean), which are expected to be distributed around zero regardless of the underlying variance. Hence, a single individual phenotype does not convey much information about the underlying variance, although differences between family groups may be substantial and estimable.

The presence of genetic heterogeneity of residual variance suggests that selection to change residual variance is possible, based mainly on family information. Thus, large families will be an advantage to study the genetic heterogeneity of variance. In fish species, both sexes are usually highly fecund, with a potential for large full- and half-sib families. The aims of this study were twofold: (1) quantify the genetic heterogeneity of (within-family) residual variance for body weight in Atlantic salmon and (2) test whether the observed heterogeneity of (within-family) residual variance can be explained by simple scaling effects. We also discuss the data structures that are best suited for estimating variance heterogeneity and individual breeding values for residual variance.

## Methods

### Phenotypic data

The analysis was restricted to year-class 2008 of Atlantic salmon from the AquaGen breeding program (http://www.aquagen.no/). Descriptive statistics of the dataset are in Table 1. Data included body weight records until harvest for 13 633 fish. Fish that were sexually mature or had visible deformities were excluded from the analysis. Potential outliers were not removed from the raw data, because this could remove the real effects that we wanted to study, e.g., occurrence of runts (undersized animals), which is a known phenomenon in Atlantic salmon.

**Table 1:** Descriptive statistics of the data

**Item**	
Number of observations (fish)	13633
Number of full-sib families	502
Number of sires	346
Number of dams	324
Number of full-sib families with paternal and/or maternal half sibs	440
Average weight, kg (SD)	3.70 (0.90)
Skewness	0.31
Kurtosis	1.25

For the production of families, both dams and sires were mated with a maximum of two partners. The final dataset included 502 full-sib families from 346 sires and 324 dams. Of these parents, 156 sires and 178 dams were represented with two full-sib offspring groups, a structure that facilitates proper separation of common (non-additive) family effects, e.g., effects due to common rearing, maternal effects and potential dominance genetic effects.

### Statistical models

The theory behind the data analysis was previously described in detail by Rönnegård et al. [12]. Both untransformed, log- and square root-transformed data were analyzed. Transformation was applied to study the potential relationships between mean and variance (scaling effects). Sire-dam models are often used in aquaculture breeding [13-15], especially for non-normal (e.g. binary) traits, and were used here to model heterogeneity of variance. In animal models, the full additive genetic effect (mid-parent mean + Mendelian sampling deviation) of each animal is fitted, whereas sire-dam models only fit the additive genetic mid-parent means (sire + dam effects) and the Mendelian sampling deviations of the offspring are included in the residuals of the model. When parents do not have own phenotypes, an animal mean model allowing for heterogeneous residual variance is equivalent to a sire-dam model in which the residual of an individual *i* is defined as [16]:

$$e_{i}^{*}=m_{i}+e_{i},$$

where *m*_*i*_ and *e*_*i*_ are, respectively, the Mendelian sampling deviation and the micro-environmental residual of individual *i*, and the variance of $e_{i}^{*}$ is thus equal to:

$$\sigma_{e_{i}^{*}}^{2}=\frac{1}{2}\sigma_{a}^{2}+\sigma_{e_{i}}^{2},$$

where $\sigma_{a}^{2}$ is the (homogeneous) additive genetic variance and $\sigma_{e_{i}}^{2}$ is the (heterogeneous) micro-environmental residual variance for animal *i* in the mean animal model. Hence, fitting an animal mean model with heterogeneous residual variance necessarily implies that the Mendelian sampling variance is assumed identical for all animals, i.e., that variance heterogeneity is strictly micro-environmental within each individual.

Because a sire-dam mean model includes the Mendelian sampling deviations into the residuals, the variance model is used to model the full within-family variance. Thus, a sire-dam variance model implies that genetic heterogeneity of residual variance in the mean model is completely explained by sire and dam effects. However, if genetic heterogeneity of variance exists, Mendelian sampling deviations are also expected to affect the level of residual variance and an animal model for the variance would be theoretically more correct. However, in our cross-sectional data (i.e. one observation per individual), these Mendelian sampling deviations only affect the variance of a single observation, i.e., some residuals will be more or less extreme than expected, but the overall effect of within-family variance on genetic heterogeneity will probably be small.

Against this background, the following models were used and compared in this study.

#### Linear mean animal model

This is a classical linear mean animal model for body weight at a given age.

y=μ+e,

(1) μ=Xb+Za+Qc,

where **y** is the vector of recorded body weights (untransformed, log-, or square root-transformed) for all fish, **b** is a vector of fixed effects (includes the effect of group of rearing at early life stages (2 groups)), **a** ~ $N\left(0,A\sigma_{a}^{2}\right)$ is a vector of additive genetic effects, **c** ~ $N\left(0,I\sigma_{c}^{2}\right)$ is a vector of common environmental family effects, **e** ~ $N\left(0,I\sigma_{e}^{2}\right)$ is a vector of random residuals, the matrices **X**, **Z** and **Q** are appropriate incidence matrices and **I** is an identity matrix of appropriate size.

#### Double hierarchical generalized linear models (DHGLM)

##### DHGLM1

The mean sub-model of the DHGLM is a classical linear mean animal model for body weight at a given age, as given above. However, the residual variance is modeled using a gamma sire-dam model, where:

(2) $$E\left(e_{i}^{2}\right)=\phi_{i},$$

$$\mathrm{Var}\left(e_{i}^{2}\right)=2\phi_{i}^{2},$$

$$\log\left(\phi_{i}\right)=X_{i}b_{D}+\left(Z_{\mathrm{Pi}}+Z_{\mathrm{Mi}}\right)\;u_{D},$$

where **b**_**D**_ is a vector of fixed effects on dispersion (equal to those for the mean model) and **u**_**D**_ is a vector of additive genetic sire and dam effects on dispersion with distribution **u**_**D **_**~**$N\left(0,A\sigma_{u}^{2}\right)$, where $\sigma_{u}^{2}=\frac{1}{4}\sigma_{a}^{2}$. The matrices **X**_**i**_, **Z**_**Pi**_ and **Z**_**Mi**_ are row incidence vectors associated with observation *i*, for the fixed, sire, and dam effects, respectively. Since the residuals are unobserved, but rather estimated as ${\hat{e}}_{i}=y_{i}-{\hat{\mu }}_{i}$, equation (2) is reformulated as:

$$E\left({\hat{e}}_{i}^{2}/\left(1-h_{i}\right)\right)=\phi_{i},$$

and can be fitted using a gamma model with weights (1-*h*_*i*_)/2, where *h*_*i*_ is the *i*^*th*^ diagonal element in the hat matrix of the mean sub-model; the hat matrix is described in Rönnegård et al. [12].

##### DHGLM2

This model is similar to the DHGLM1 described above, except that the additive genetic animal effect in the mean model is replaced by additive genetic effects of the sire and the dam (as in the variance model):

$$\mu =X_{b}+\left(Z_{P}+Z_{m}\right)\;u+Qc,$$

where **u** is a vector of additive genetic effects (on the mean) of the sire and the dam.

Restricted maximum likelihood methods were used to estimate variance components with the ASREML software [17]. For the DHGLM models, an iterative approach within the R environment (http://www.r-project.org), alternating between weighted analyses of the mean and variance sub-models was used, with weights being updated for each round, as described in Rönnegård et al. [12].

## Results

Variance components estimated based on the different models are in Table 2 for the untransformed data, in Table 3 for the log-transformed data and in Table 4 for the square root-transformed data. The linear mean animal model revealed considerable amounts of additive genetic variance for body weight in Atlantic salmon, with a genetic variance of 0.30 kg^2^ and a heritability of 0.36 ± 0.04 (0.32 ± 0.04 and 0.35 ± 0.04 for log- and square root-transformed data, respectively). However, for both the original and transformed data, the two DHGLM models deviated considerably from each other. The DHGLM2 (sire-dam model) and the classical linear mean animal model gave similar estimates of genetic (after appropriate rescaling) and common environmental variances on the mean, whereas the DHGLM1 (animal/sire-dam model) produced a considerably lower (~ −70%) estimate of genetic variance and a much larger (~ +300%) estimate of common environmental variance on the mean. With the linear mean animal model (ignoring heterogeneity of within-family variance), genetic variance is estimated based on covariance between relatives. Hence, even in the presence of genetic heterogeneity of within-family variance, the mean model is expected to produce unbiased estimates of the genetic variance on the mean level, which was also confirmed in a preliminary simulation study (results not shown). Since the linear mean animal model is expected to give unbiased estimates of genetic variance (on the mean), the discrepancy between estimates from the linear mean animal model and the DHGLM1 indicates that the latter model is substantially biased, while the DHGLM2 appears appropriate.

**Table 2:** Variance components of the classical linear mean animal model and two double hierarchical generalized linear models DHGLM1 and DHGLM2 (untransformed data)

**Sub-model**	**Variance component**	**Classical**	**DHGLM1**	**DHGLM2**
Mean	Sire-dam	-	-	0.071 ± 0.010
	Genetic^1^	0.303 ± 0.041	0.099 ± 0.023	0.283 ± 0.039
	Common environment	0.013 ± 0.008	0.063 ± 0.010	0.016 ± 0.008
	Residual	0.521 ± 0.022	-	-
	Heritability	0.362 ± 0.043	-	-
Variance	Sire-dam	-	0.051 ± 0.008	0.043 ± 0.008
	Genetic^1^	-	0.204 ± 0.033	0.174 ± 0.031

^1^Genetic variance is defined as 4*sire-dam variance.

**Table 3:** Variance components of the classical linear mean model and two double hierarchical generalized linear models DHGLM1 and DHGLM2 (log-transformed data)

**Sub-model**	**Variance component**	**Classical**	**DHGLM1**	**DHGLM2**
Mean	Sire-dam	-	-	0.005 ± 0.001
	Genetic^1^	0.023 ± 0.003	0.007 ± 0.002	0.022 ± 0.003
	Common environment	0.001 ± 0.001	0.005 ± 0.001	0.001 ± 0.001
	Residual	0.047 ± 0.002	-	-
	Heritability	0.321 ± 0.040	-	-
Variance	Sire-dam	-	0.042 ± 0.011	0.034 ± 0.009
	Genetic^1^	-	0.166 ± 0.042	0.138 ± 0.037

^1^Genetic variance is defined as 4*sire-dam variance.

**Table 4:** Variance components of the classical linear mean model and two double hierarchical generalized linear models DHGLM1 and DHGLM2 (root-transformed data)

**Sub-model**	**Variance component**	**Classical**	**DHGLM1**	**DHGLM2**
Mean	Sire-dam	-	-	0.019 ± 0.003
	Genetic^1^	0.082 ± 0.011	0.030 ± 0.007	0.078 ± 0.011
	Common environment	0.003 ± 0.002	0.016 ± 0.003	0.004 ± 0.002
	Residual	0.149 ± 0.006	-	-
	Heritability	0.350 ± 0.042	-	-
Variance	Sire-dam	-	0.040 ± 0.008	0.033 ± 0.007
	Genetic^1^	-	0.160 ± 0.032	0.133 ± 0.028

^1^Genetic variance is defined as 4*sire-dam variance.

For the variance sub-model, the estimated sire-dam genetic variances from DHGLM2 for the untransformed and transformed data were 0.04 ± 0.01 and 0.03 ± 0.01 (log- and square root), respectively. The sire-dam variance component is ¼ of the total genetic variance $\left(\sigma_{a}^{2}\right)$ for the trait (sire and dam combined explain ½ $\sigma_{a}^{2}$). Thus, the results obtained with DHGLM2 for all transformations indicate that genetic variance in canalization exists, which implies that families differed in their sensitivity to environmental (and potentially genetic) changes. The importance of the genetic heterogeneity of within-family variance can be illustrated by the following example. Assuming a population with an average within-family variance for body weight of 0.67 (as in the current untransformed dataset), equation (2) implies an expectation on the log scale of *μ* = −0.40. A log-scale 95% prediction interval for mid-parent variance breeding values, i.e. the interval for which 95% of future families is expected to fall in absence of selection, can then be defined as $\left[\mu -1.96\sqrt{\frac{1}{2}\sigma_{a}^{2}},\mu +1.96\sqrt{\frac{1}{2}\sigma_{a}^{2}}\right]=\left[-0.98,-0.18\right]$, which corresponds to within-family variances of body weight in the range [*e*^− 0.98^, *e*^− 0.18^] = [0.38, 1.18]. This shows that the variation of the most extreme high families is expected to be approximately three times larger than the extreme low families.

Pearson correlation coefficients between estimated breeding values for the mean and variance sub-models using DHGLM2 were all significantly different from zero, but differed substantially between transformations (0.42, -0.17 and 0.14 for untransformed, log-transformed and square root transformed data, respectively). Thus, transformation of the data makes estimated breeding values for the mean and variance more independent. This may also explain the somewhat reduced genetic variance of the variance model after transformation.

## Discussion

The main result of this study is that substantial heterogeneity of within-family variance exists for body weight in Atlantic salmon. Furthermore, the iterative approach revealed that the DHGLM animal mean model (DHGLM1) resulted in severely biased estimates of variance components when employed with cross-sectional data, both when compared with a linear mean animal model and a sire-dam DHGLM (DHGLM2) model.

### Statistical models

Since genetic heterogeneity of variance exists between families, an equivalent genetic heterogeneity must also exist within families. Hence, an animal model should ideally be fitted in both the mean and the variance sub-models of the DHGLM. This would be of particular importance for datasets with repeated records on individuals, and such data structures would allow proper estimation of variances on an individual level. However, the current dataset contained cross-sectional data (one observation per individual), which implies that the variance cannot be estimated on the individual level but only for groups of individuals (families). This may explain why any attempt to model the variance using an animal model did not converge, possibly due to the iterative approach used, where one iterates between the mean and variance sub-models and the models are thus allowed to interact. In earlier studies, more approximate non-iterative methods have been used [7,10,18], where a linear mean animal model is fitted first and the variance model is subsequently applied to the (log of) squared residuals. For these non-iterative methods (with fixed variance “phenotypes” coming from a linear mean model), animal variance models could be fitted. For the more advanced iterative approaches, more research is needed on the optimization of data structures with respect to proper identification of individual breeding values for the variance.

Since genetic variance in the classical linear mean animal model is estimated based on between-family variation in observed mean phenotypes, the estimated genetic variance is expected to be unbiased. Likewise, the estimated residual variance is expected to reflect the typical level of within-family variation. This would be the case even if the true underlying model includes genetic heterogeneity of residual variance, which was confirmed in a simulation study with 200 families of 40 individuals resulting from a perfect 2 sires × 2 dams mating system of 100 sires and 100 dams (results not shown). In principle, the DHGLM models are expected to give similar genetic variance estimates on the mean as the linear mean animal model, because these estimates are also based on observed between-family variation. Hence, in the real data analysis, the mean sub-models of the two DHGLM models are expected to give similar results as a linear mean animal model. This was indeed the case for the DHGLM2 model but the DHGLM1 model showed substantial deviations. Hence, the results of this study indicate that the DHGLM1 model is severely biased when the data does not contain repeated records. The two DHGLM models differed in the flexibility of the variance sub-model; in the DHGLM1 model, the Mendelian sampling variance is assumed homogeneous (and restricted to $\frac{1}{2}\sigma_{a}^{2}$), while DHGLM2 includes the Mendelian sampling effects in the residuals, and the entire within-family variance is modeled by the variance sub-model, allowing for heterogeneity. The latter is consistent with the concept of canalization, which leads to genetic heterogeneity of variance due to varying sensitivities to potentially both environmental and genetic perturbations [1]. If sensitivities to both environmental and genetic perturbations contribute to the observed heterogeneity of variance, the underlying assumption of DHGLM1 is violated while DHGLM2 would still be appropriate. Our initial hypothesis was therefore that the bias of the DHGLM1 model was due to an incorrect assumption of homogeneous Mendelian sampling variance. To test this, we fitted the DHGLM1 and DHGLM2 models to simulated cross-sectional data (as described above), using either homogeneous (as in DHGLM1) or heterogeneous Mendelian sampling variances (appropriate for DHGLM2); the residual (micro-environmental) variance was always heterogeneous, which is consistent with both models. The results were striking: the DHGLM1 model always resulted in biased estimates of the variance components (similar to the real data analysis), even when the Mendelian sampling variance was homogeneous, while DHGLM2 was unbiased in both cases (results not shown). Hence, DHGLM1 was biased even when its assumptions were true. Furthermore, we found that the bias was restricted to statistical models in which both animal additive genetic and common environmental family effects were fitted in the mean sub-model, i.e., when between-family variance on the mean can potentially be explained by common environmental factors. Thus, it appears that DHGLM models that include random family-specific effects (genetic and/or common environment) in the variance sub-model will use these to describe as much of the within-family variance as possible. This would be appropriate for sire-dam mean sub-models (since the entire within-family variance is included in the residual variance) but is not appropriate for animal mean sub-models (since Mendelian deviations are included in the additive genetic effects). Again, such bias is more likely observed in iterative methods such as those of Rönnegård et al. [12], which allow the mean and variance sub-models to interact with each other. However, a non-iterative procedure, like the one applied in [7,10,18], should not be used without critically assessing the assumptions [19], because there is a risk of drawing conclusions from data that contains no (or very little) information to infer genetic heterogeneity of the residual variance.

We have chosen to quantify the genetic heterogeneity in terms of variance components rather than heritability because variance components in the variance sub-model are easier to interpret and are comparable across experiments and species (when an exponential model for the variance is used). For instance, a genetic variance of 0.17 in the variance model, as found for DHGLM2, can be expressed as a standard deviation of 0.41 (i.e. the square-root of 0.17), which implies that a unit standard deviation increase/decrease of the breeding value for the variance increases/decreases the residual variance by 51% and 36%, respectively, because the model is multiplicative and can be shown to be [9]:

$$\sigma_{e-\mathrm{new}}^{2}=\sigma_{e-\mathrm{old}}^{2}.\exp\left(\Delta G_{v}\right),$$

where $\sigma_{e-\mathrm{old}}^{2}$ is the residual variance prior to selection, $\sigma_{e-\mathrm{new}}^{2}$ is the residual variance after selection and *ΔG*_*v*_ is the genetic gain on the underlying log scale of the variance. Hence, the genetic standard deviation of residual variance is an informative measure for two reasons: (1) it gives an idea of how large the proportional change in the residual variance would be for a unit standard deviation increase/decrease of the residual variance breeding values and (2) this proportional change does not depend on the phenotypic variance and can therefore be compared across experiments and species. The value of using the genetic standard deviation of residual variance was emphasized already in Mulder et al. [11] where they refer to it as the genetic coefficient of variation. An alternative would be to use the Mulder-Hill heritability for the residual variance, i.e. the regression of the variance breeding values on squared phenotype values [11], but this measure is model-specific and not as easy to interpret.

Common environmental effects were not included in the variance sub-models. The main reason for this is that genetic and common environmental effects are difficult to disentangle, and more so with respect to the variance model, since there is only one variance group per family. In contrast, data with repeated records per individual will have one variance group per animal rather than one per family. In our case, the data structure makes the separation between genetic and common environment factors on the variance scale difficult, which was confirmed in the simulation study described above, where the DHGLM2 model was extended with a common environmental family effect in the variance sub-model. Although all the simulated heterogeneity of variance was due to genetics, the model distributed the dispersion effects more or less randomly over genetic and common environmental factors (varying between replicates). This confirms the low power of separating such effects in the variance model, despite a perfect mating system of 2 sires × 2 dams. Furthermore, a preliminary analysis of the real data also revealed that partitioning additive genetic and common environmental effects in the variance model was sensitive to transformation of data, while the mean model was rather robust in this regard. Judging by the results of the linear animal mean model, the common environmental effects were small and not very significant (likelihood ratio test, P > 0.05, results not shown). Still, we cannot rule out the existence of common environmental effects acting on the variance, and if they exist, the estimated genetic variance of the variance sub-model is probably inflated.

It may be argued that heterogeneity of within-family variances may, to some extent, be explained by simple scaling effects, i.e., variance may be a function of the expected mean. If this is the case, larger genetic and environmental variances should be expected for families with higher mean body weight. Yang et al. [20] studied the effect of (Box-Cox) transformation of the data to stabilize variances. In one dataset (rabbits), evidence for genetic heterogeneity was weaker after transformation, while the opposite was true for other datasets (pigs). Based on the moderately positive correlation between estimated parental breeding values from the mean and variance models for untransformed data (DHGLM2) of the current dataset, scaling effects may explain some of the genetic heterogeneity of within-family variance. The phenotypic data was therefore also log-transformed to remove a potential relationship between mean and variance. This reduced but did not remove heterogeneity of within-family variances (Table 3). Both log and untransformed data are extremes of the Box-Cox transformation (corresponding to λ = 0 and λ = 1, respectively). Therefore, an intermediate transformation (square root, equivalent to λ = 0.5) was also tested but gave similar results to those of the log-transformation (Table 3). Hence, our results indicate substantial heterogeneity of (within-family) variance for body weight in Atlantic salmon, apart from what is expected from simple scaling effects.

### Implications for aquaculture breeding

The results of this study indicate that there is genetic heterogeneity of (within-family) variance for body weight in Atlantic salmon. Selection for decreased within-family variance may give more homogeneous stocks, which would be an advantage in commercial fish farming. Increased homogeneity is consistent with the concept of canalization, but according to theory, canalization may be due to varying sensitivity to both environmental and genetic perturbations, i.e., the Mendelian sampling variance may also differ between families. Our study does not prove the presence of heterogeneity of Mendelian sampling variance, nor does it rule out the potential existence of such effects. A complication for selection for increased homogeneity based on cross-sectional data, which is by far the most common data structure in aquaculture, is that the Mendelian sampling and micro-environmental residual effects are completely confounded, which implies that pure selection against micro-environmental sensitivity cannot be guaranteed.

Genetic heterogeneity in sensitivity to micro-environmental change (on the individual scale) is one form of genotype by environment interaction. However, genotype by environment interactions may also be present on the macro-environmental scale (diet, climatic region, etc.), potentially causing re-ranking among families. For example, some families may have highly variable phenotypes for one diet but more homogeneous phenotypes for another diet. Hence, one may expect substantial re-ranking of estimated breeding values for variance among cohorts. Such factors would complicate meaningful selection for altered environmental sensitivity in practical breeding programs aimed at providing genetic material for production systems over a wide range of macro-environments.

## Conclusions

Substantial heterogeneity of within-family variance was found for body weight in Atlantic salmon, with a 95% prediction interval for within-family variance ranging from ~0.4 to ~1.2 kg^2^, showing that the variation of the most extreme high families is approximately three times larger than the extreme low families. A tendency towards higher variance with higher means (scaling effects) was observed, but heterogeneity of within-family variance existed beyond what could be explained by simple scaling effects. Since there were no repeated observations, the fitting algorithm biased the estimates of the variance components of the DHGLM animal mean model (DHGLM1), but not of a classical linear mean animal model and a pure sire-dam DHGLM (DHGLM2) model. However, selection for increased homogeneity of variance may still prove difficult, especially when based on cross-sectional data. Furthermore, potential macro-environmental changes may make genetic heterogeneity of variance a less stable trait over time and space.

## Competing interests

The authors declare that they have no competing interest.

## Authors’ contributions

AKS initiated the study. JØ analyzed the data. The manuscript was drafted by AKS and JØ. LR provided the necessary R scripts for the analyses. All authors read and approved the final manuscript.

## References

[B1] Waddington CH Canalization of development and the inheritance of acquired characters Nature 1942 45 563 565 10.1038/150563a0 13666847

[B2] SanCristobal-Gaudy M Bodin L Elsen JM Chevalet C Genetic components of litter size variability in sheep Genet Sel Evol 2001 45 249 271 10.1186/1297-9686-33-3-249 11403747 PMC2705407

[B3] Sorensen D Waagepetersen R Normal linear models with genetically structured residual variance heterogeneity: a case study Genet Res 2003 45 207 222 10.1017/S0016672303006426 15134199

[B4] Gutiérrez J Nieto B Piqueras P Ibáñez N Salgado C Genetic parameters for canalisation analysis of litter size and litter weight traits at birth in mice Genet Sel Evol 2006 45 445 462 10.1186/1297-9686-38-5-445 16954039 PMC2689256

[B5] Ibáñez-Escriche N Sorensen D Waagepetersen R Blasco A Selection for environmental variation: A statistical analysis and power calculations to detect response Genetics 2008 45 2209 2226 10.1534/genetics.108.091678 18832357 PMC2600953

[B6] Damgaard LH Rydhmer L Løvendahl P Grandinson K Genetic parameters for within-litter variation in piglet birth weight and change in within-litter variation during suckling J Anim Sci 2003 45 604 610 12661639 10.2527/2003.813604x

[B7] Mulder HA Hill WG Vereijken A Veerkamp RF Estimation of genetic variation in residual variance in female and male broiler chickens Animal 2009 45 1673 1680 10.1017/S1751731109990668 22443551

[B8] Hill WG Mulder HA Genetic analysis of environmental variation Genet Res 2010 45 381 395 10.1017/S0016672310000546 21429270

[B9] Rönnegård L Felleki M Fikse WF Mulder HA Strandberg E Variance component and breeding value estimation for genetic heterogeneity of residual variance in Swedish Holstein dairy cattle J Dairy Sci 2013 45 2627 2636 10.3168/jds.2012-6198 23415533

[B10] Janhunen M Kause A Vehviläinen H Järvisalo O Genetics of microenvironmental sensitivity of body weight in rainbow trout (*Oncorhynchus mykiss*) selected for improved growth PLoS One 2012 45 e38766 10.1371/journal.pone.0038766 22701708 PMC3372501

[B11] Mulder HA Bijma P Hill WG Prediction of breeding values and selection responses with genetic heterogeneity of environmental variance Genetics 2007 45 1895 1910 10.1534/genetics.106.063743 17277375 PMC1855112

[B12] Rönnegård L Felleki M Fikse F Mulder HA Strandberg E Genetic heterogeneity of residual variance - estimation of variance components using double hierarchical generalized linear models Genet Sel Evol 2010 45 8 10.1186/1297-9686-42-8 20302616 PMC2859745

[B13] Ødegård J Olesen I Dixon P Jeney Z Nielsen H-M Way K Joiner C Jeney G Ardó L Rónyai A Gjerde B Genetic analysis of common carp (*Cyprinus carpio*) strains. II: Resistance to koi herpesvirus and Aeromonas hydrophila and their relationship with pond survival Aquaculture 2010 45 7 13 10.1016/j.aquaculture.2010.03.017

[B14] Ødegård J Olesen I Gjerde B Klemetsdal G Evaluation of statistical models for genetic analysis of challenge test data on furunculosis resistance in Atlantic salmon (*Salmo salar*): Prediction of field survival Aquaculture 2006 45 116 123 10.1016/j.aquaculture.2006.05.034

[B15] Drangsholt TM Gjerde B Ødegård J Finne-Fridell F Evensen O Bentsen HB Quantitative genetics of disease resistance in vaccinated and unvaccinated Atlantic salmon (*Salmo sala*r *L*.) Heredity 2001 45 471 477 10.1038/hdy.2011.34 PMC3199929 21559049

[B16] Ødegård J Meuwissen THE Heringstad B Madsen P A simple algorithm to estimate genetic variance in an animal threshold model using Bayesian inference Genet Sel Evol 2010 45 29 10.1186/1297-9686-42-29 20649962 PMC2918534

[B17] Gilmour AR Gogel BJ Cullis BR Thompson R ASReml user guide release 3.0 2009 VSN International Ltd: Hemel Hempstead

[B18] Neves HR Carvalheiro R Queiroz SA Genetic and environmental heterogeneity of residual variance of weight traits in Nellore beef cattle Genet Sel Evol 2012 45 19 10.1186/1297-9686-44-19 22672564 PMC3390267

[B19] Rönnegård L Valdar W Recent developments in statistical methods for detecting genetic loci affecting phenotypic variability BMC Genet 2012 45 63 22827487 10.1186/1471-2156-13-63 PMC3493319

[B20] Yang Y Christensen OF Sorensen D Analysis of a genetically structured variance heterogeneity model using the Box–Cox transformation Genet Res 2011 45 33 46 10.1017/S0016672310000418 21349235

